# Quantum Dots for Multiplexed Detection and Characterisation of Prostate Cancer Cells Using a Scanning Near-Field Optical Microscope

**DOI:** 10.1371/journal.pone.0031592

**Published:** 2012-02-09

**Authors:** Kelly-Ann D. Walker, Claire Morgan, Shareen H. Doak, Peter R. Dunstan

**Affiliations:** 1 College of Engineering, Multidisciplinary Nanotechnology Centre, Swansea University, Singleton Park, Swansea, United Kingdom; 2 Institute of Life Science, College of Medicine, Swansea University, Singleton Park, Swansea, United Kingdom; 3 College of Science, Dept of Physics, Multidisciplinary Nanotechnology Centre, Swansea University, Singleton Park, Swansea, United Kingdom; George Mason University, United States of America

## Abstract

In this study scanning near-field optical microscopy (SNOM) has been utilised in conjunction with quantum dot labelling to interrogate the biomolecular composition of cell membranes. The technique overcomes the limits of optical diffraction found in standard fluorescence microscopy and also yields vital topographic information. The technique has been applied to investigate cell-cell adhesion in human epithelial cells. This has been realised through immunofluorescence labelling of the cell-cell adhesion protein E-cadherin. Moreover, a dual labelling protocol has been optimised to facilitate a comparative study of the adhesion mechanisms and the effect of aberrant adhesion protein expression in both healthy and cancerous epithelial cells. This study reports clear differences in the morphology and phenotype of healthy and cancerous cells. In healthy prostate epithelial cells (PNT2), E-cadherin was predominantly located around the cell periphery and within filopodial extensions. The presence of E-cadherin appeared to be enhanced when cell-cell contact was established. In contrast, examination of metastatic prostate adenocarcinoma cells (PC-3) revealed no E-cadherin labelling around the periphery of the cells. This lack of functional E-cadherin in PC-3 cells coincided with a markedly different morphology and PC-3 cells were not found to form close cell-cell associations with their neighbours. We have demonstrated that with a fully optimised sample preparation methodology, multiplexed quantum dot labelling in conjunction with SNOM imaging can be successfully applied to interrogate biomolecular localisation within delicate cellular membranes.

## Introduction

Cellular adhesion plays an important role in maintaining the architecture of tissues and organs and is vital for their correct functioning. Abnormal cell-cell adhesion has implications in the onset of many illnesses and diseases. A concerted effort has been made by many researchers to determine the relationship between cellular adhesion and the metastatic capacity of many cancers [1], [2].

E-cadherin is one of the principle mediators of cell-cell adhesion in epithelial tissues and has been extensively examined to determine its role in cancer progression and metastasis. It has been demonstrated that the loss of E-cadherin expression or function is linked to increased invasive potential [3], metastatic potential [4] and poorer patient prognosis [5], [6]. This relationship is particularly relevant to prostate cancers which have a propensity to metastasise and form secondary tumours (primarily skeletal), resulting in a poor patient prognosis [7], [8]. Although significant efforts have been made towards understanding the role of E-cadherin in the progression of prostate cancer, researchers have not reached a consensus [9], [10]. A more detailed understanding of adhesion mechanisms could lead to the development of novel cancer treatments as indicated by the preliminary studies carried out by Zhou *et al.* [11] and Mao *et al.* [12].

**Figure 1:**
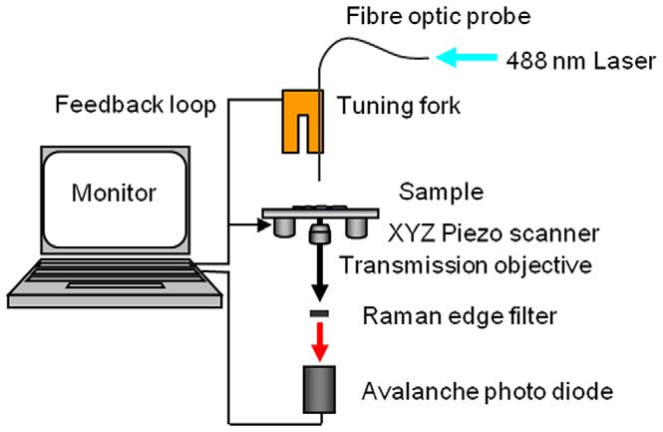
Schematic of the SNOM experimental arrangement.

Although routinely applied across the life sciences, conventional fluorescence microscopy techniques are limited by the optical diffraction limit. Accessing information beyond the diffraction limit from a wide range of sample types has been made possible following the emergence of scanning probe microscopy (SPM) techniques. SPM enables examination of a sample's metrology with nanoscale resolution and scanning near-field optical microscopy (SNOM) is one such example which is particularly suited to interrogate the interactions and functions of biological materials [13]–[15]. SNOM has the capacity to simultaneously probe topography and examine optical features on scales that can not normally be achieved using conventional fluorescence microscopy by exploiting the properties of evanescent waves. A typical SNOM experimental set-up is illustrated in Figure 1. Whilst there have been rapid advances (see the review by Galbraith and Galbraith [16]) in the field of super-resolution optical microscopy with the development of techniques such as stimulated emission depletion (STED), photo-activated localisation microscopy (PALM) and fluorescence imaging with one-nanometer accuracy (FIONA) [17], the use of optical near-fields for surface or membrane investigations using total internal reflection fluorescence microscopy (TIRFM) has also become more commonplace [18], [19]. By its nature, TIRFM restricts the illuminated Z-range and hence offers better resolution than confocal microscopy. SNOM has the benefits of TIRFM but in addition generates an optical near-field which is also spatially confined to nanometre dimensions in x and y. Moreover its probe is combined with a topographic feedback mechanism that allows it to simultaneously reveal the structural changes of the sample alongside its optical response.

This paper reports the use of an optimised dual labelling protocol to lead a comparative study of the adhesion mechanisms in both healthy and cancerous epithelial cells. The focus of the study has been a high resolution examination using SNOM on the function of the E-cadherin protein in two cell lines. As well as E-cadherin, the tight junction protein ZO-1 was selected as a suitable imaging control as it is expressed in the plasma membrane at the cell-cell boundary. Thus, antibodies against the adhesion proteins E-cadherin and ZO-1 were utilised to indirectly label normal prostate epithelial cells PNT2 and prostate adenocarcinoma cells PC-3. The SNOM technique examines the differential sub-cellular localisation of cell-cell adhesion molecules at high resolution and has been performed in parallel with gene expression studies to give overall indications on both function and expression.

## Materials and Methods

### Cell Culture

Prostate epithelial cells, PNT2, and prostate adenocarcinoma cells, PC-3, were obtained from the European Collection of Cell Cultures (Salisbury, UK). The cells were maintained in RPMI 1640 (Gibco, Paisley, UK) supplemented with 10% fetal bovine serum, 1% L-Glutamine, 60 units/mL penicillin and 60 µg/mL streptomycin at 37°C/5% CO_2_. The cells were sub-cultured when they reached approximately 80% confluency.

For imaging, cells were grown on sterile 25 mm circular glass coverslips. The cells were fixed at room temperature using 3.7–4% ultra-pure methanol-free formaldehyde (Park Scientific Ltd., Northampton, UK) in PBS for 15 minutes, followed by three 5 minute washes in PBS/100 mM Glycine and dehydrated through an ethanol series. Samples were stored at 4°C until required for fluorescence labelling.

### RNA extraction and Real-time polymerase chain reaction (PCR)

Cells were grown to confluence in 75 cm^3^ flasks. Total cellular RNA was extracted using the RNeasy extraction kit (Qiagen, Crawley, UK) and residual DNA was removed by treating with DNA-free™ (Ambion, Cambridgeshire, UK) according to the manufacturer's instructions. cDNA was synthesised from 1 µg RNA using random primers and the High Capacity cDNA synthesis reverse transcription kit (Applied Biosystems, Warrington, UK). Real-time PCR reactions were performed in the iCycler iQ Thermal Cycler (Bio-Rad laboratories, Hemel Hempstead, UK) using the SYBR Green detection methodology. Primer sets were designed to be exon-exon spanning in order to minimise the possibility that any contaminating genomic DNA would be amplified. The primer sets used were also tested to ensure they all demonstrated equal amplification efficiencies. All reactions were performed in triplicate with 2 µl cDNA, 12.5 µl iQ SYBR Green Supermix (Bio-Rad laboratories, Hemel Hempstead, UK) and 0.2 µM forward and reverse primers, in a final reaction volume of 25 µl. PCR amplification conditions were 95°C for 3 minutes, followed by 40 cycles of 94°C for 30 seconds, 60°C for 30 seconds and 72°C for 30 seconds. Fold expression was normalised to PNT2. E-cadherin and ZO-1 were amplified using the primers shown in Table 1. Also shown are the primer sequences for β-actin and HPRT, which were used as endogenous controls.

**Table 1:** Primer Sequences.

Gene	Forward Primer (5′-3′)	Reverse Primer (5′-3′)
E-cadherin	CTGTCGAAGCAGGATTGCAAA	GAAGAAACAGCAAGAGCAGCA
ZO-1	GGGAGGGTGAAGTGAAGACA	GATCTGAAGSGGCCATGGAA
β-actin	GATGGCCACGGCTGCTTC	TGCCTCAGGGCAGCGGAA
HPRT	GACTGTAGATTTTATCAGACTGA	TGGATTATACTGCCTGACCAA

### Western blotting

Total protein was extracted using RIPA buffer (Sigma Aldrich, Dorset, UK). Thirty micrograms of protein was run on a 7.5% tris-glycine PAGE gels (Bio-Rad laboratories, Hemel Hempstead, UK). Proteins were transferred onto nitrocellulose membrane (Amersham Pharmacia Biotech, Amersham, UK). The membranes were blocked with 5% non fat dry milk in tween/TBS (all Sigma Aldrich, Dorset, UK) to inhibit non specific binding and incubated overnight at 4°C using mouse anti-ZO-1 antibody (Invitrogen, Paisley, UK) at 1∶250 and rabbit anti-E-cadherin antibody (New England Biolabs, Hertfordshire, UK) at 1∶1000. β-actin (1∶1000) was used as a control for protein loading. Membranes were washed free of primary antibody and incubated with horseradish peroxidase conjugated secondary anti-mouse/anti-rabbit antibodies (1∶1000) for 1 hour at room temperature. Proteins were visualized using the Immun-Star WesternC chemiluminescene detection kit (Bio-Rad laboratories, Hemel Hempstead, UK) and quantified using densitometry and Quantity One software (Bio-Rad laboratories, Hemel Hempstead, UK).

### Immunofluorescence

All stages were performed under ambient conditions unless otherwise stated. Immunolabelling was generally carried out within 24 hours of fixation. All steps of the immunofluorescence labelling protocols were optimised from the basic procedures described by the manufacturers; including permeabilisation conditions, blocking steps, concentration of reagents, incubation conditions and washing steps. The optimisation was completed for each cell line used within this study. Negative control experiments were carried out alongside fluorescence labelling to ensure selectivity of the fluorescent labels. Negative controls were not found to produce any distinct labelling pattern or to have any significant fluorescence yield. To dual label PNT2 cells against E-cadherin and ZO-1, the coverslips were rinsed with PBS, permeabilised with 0.1% Triton-X for 10 minutes and incubated with Image-iT FX signal enhancer (Invitrogen, Paisley, UK) for 30 minutes. The coverslips were then rinsed and subsequently incubated with 31 µg/ml mouse anti-ZO-1 antibody (Invitrogen, Paisley, UK) for 2 hours at 37°C. To label ZO-1 with quantum dots, the coverslips were incubated with 30 nM Qdot 525 goat anti-mouse IgG conjugate (Invitrogen, Paisley, UK) for 1 hour. Rabbit anti-E-cadherin primary antibody (Abcam, Cambridge, UK) was then applied to the cells at a concentration of 18 µg/ml and coverslips were incubated for 2 hours at 37°C. To label E-cadherin with quantum dots, Qdot 605 goat anti-rabbit was applied at a concentration of 20 nM and left for 1 hour (Invitrogen, Paisley, UK). Prior to imaging, the coverslips were rinsed in PBS to remove excess unbound quantum dots. To facilitate SNOM imaging, samples were additionally rinsed in water, dehydrated through an ethanol series and air-dried. For dual labelling of PC-3 cells a slightly different protocol was required. To reduce background staining, coverslips were blocked with PBS/100 mM Glycine prior to the application of antibodies and the order of application was E-cadherin primary antibody, Qdot 605 goat anti-rabbit antibody, ZO-1 primary antibody and Qdot 525 goat anti-mouse secondary antibody (20 nM concentration was used for Qdot 525, all other concentrations and incubation times were as for PNT2 cells). Samples were observed using fluorescence microscopy prior to SNOM imaging to verify that labelling had been successful. Samples were imaged within 48 hours of preparation.

### SNOM imaging

The SNOM images were obtained using an Aurora 3 (Veeco Instruments, Cambridge, UK), using aluminium coated fibre optic probes with resonant frequency 80–110 kHz and aperture of approximately 50–100 nm, as specified by the manufacturers (Veeco Instruments, Cambridge, UK). A 488 nm Ar^+^ laser was used for excitation and transmission signals were collected with a 40×, 0.65 NA collection objective. The signals were passed through a 488 nm Raman edge (high pass) optical filter to eliminate light from the laser. To image the location of ZO-1 proteins (labelled with green emitting quantum dots), the remaining light was passed through a 525 nm optical band pass filter, to remove red fluorescence, and focussed onto an avalanche photo diode (APD). To image the location of E-cadherin proteins (labelled with red emitting quantum dots), a 609 nm optical band pass filter was utilised. In this article the APD signals which arise due to fluorescence are presented and typical APD integration times were 20 ms/pixel, with image resolution of 300×300 pixels.

### Statistical analysis

To test the assumption that data were normally distributed, normal plots were performed for each group. One sample Kolmogorov-Smirnov statistics resulted in p values >0.05 indicating data was normally distributed. Analysis was therefore carried out using the parametric ANOVA test. Dunnett post hoc analysis was performed to compare cancer cell lines with the non-cancerous cell line control and Tukey post hoc analysis was performed for multiple group comparisons. A *p*-value <0.05 was considered significant.

## Results

### Expression Analysis

**Figure 2:**
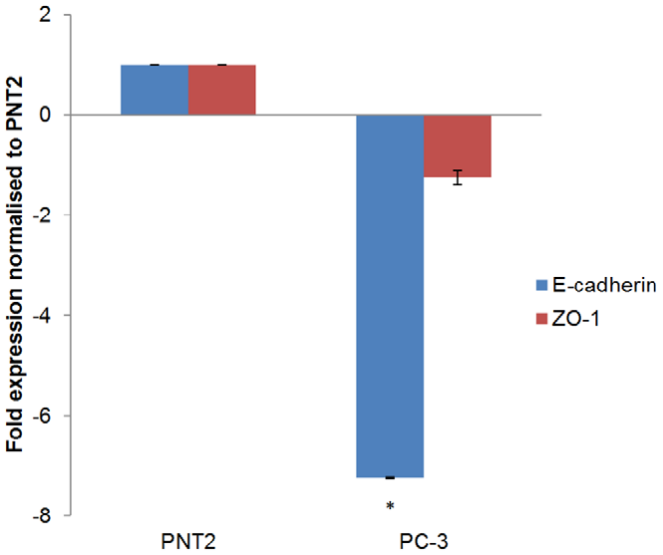
Differential mRNA expression of E-cadherin and ZO-1. Expression levels have been normalised against two housekeeping genes and change in expression relative to PNT2 is illustrated. * denotes significant difference (P<0.05) compared to PNT2.

Gene expression data was obtained through real-time polymerase chain reaction (PCR) and for E-cadherin, revealed a statistically significant decrease (−7.25 fold) in expression in PC-3 (*p* = <0.001) compared to PNT2. For ZO-1, even though expression was down regulated in PC3 in comparison to PNT2 (−1.20 fold) this was not deemed significant (Figure 2).

**Figure 3:**
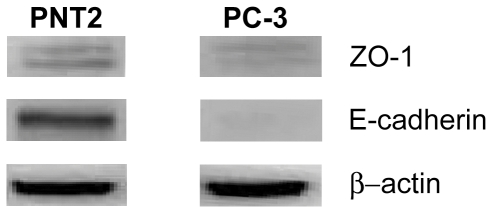
Western blot comparing the metastatic prostate cancer cell line PC-3 to normal prostate epithelium (PNT2). A significant down regulation of E-cadherin is observed whilst differences in the ZO-1 protein expression between the cell lines is less significant. Note, β-actin was used for a loading control.

Western blot analysis revealed that down regulation of E-cadherin was also evident at the protein level. Densitometry revealed a 2.6 fold down regulation of E-cadherin in the PC-3 cell line compared to PNT2. The protein levels for ZO-1 were also down regulated (−1.05 fold) in relation to PNT2, supporting the gene expression data (Figure 3).

### Dual labelled PNT2 cells

**Figure 4:**
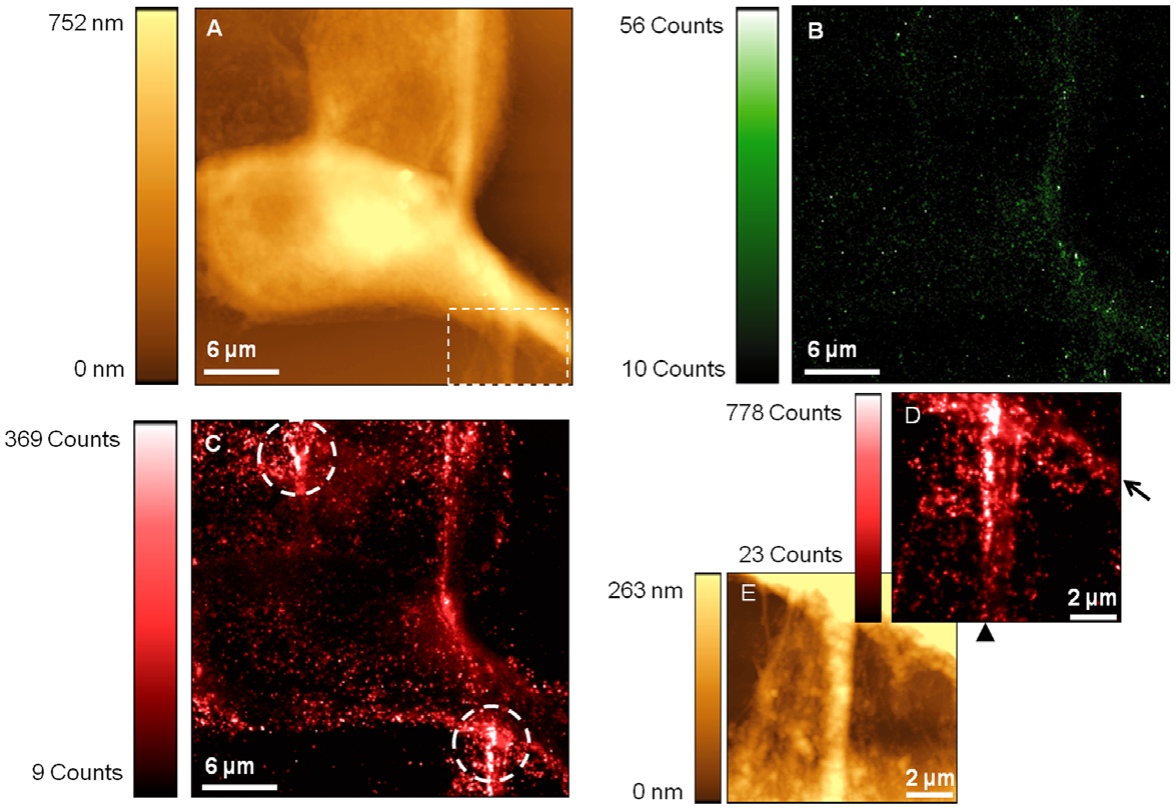
SNOM acquisitions of dual labelled PNT2 cells. Images reveal the topography (A and E) and fluorescence (B, C and D) response. Images (B) and (C) were obtained with different filters to spectrally distinguish between red and green fluorescence, identifying ZO-1 and E-cadherin locations respectively. The boxed region in (A) was scanned at higher resolution to generate the detailed E-cadherin fluorescence image (D) and the accompanying topography is shown in (E). Circled regions in (C) and the arrow/arrowhead in (D) highlight E-cadherin clusters.

To achieve filter separation of the fluorescence signals produced by the dual labelled samples, it was important to utilise quantum dots which emit at distinctly separated wavelengths, hence the selection of quantum dots with emission wavelengths of 605 nm and 525 nm. PNT2 samples were examined using SNOM and the images presented in Figure 4 were generated. Topography information can be seen in Figure 4A, which was acquired to demonstrate that the extensive dual labelling protocol did not result in changes to the structure of the sample. As can be seen in Figure 4A, the cellular structure remained well preserved and features can clearly be distinguished.

Corresponding optical images were acquired for the topography image shown in Figure 4A to reveal the location of ZO-1 and E-cadherin (shown in Figures 4B and 4C respectively). Gene expression analysis revealed ZO-1 is expressed in both cell lines and so for the purposes of this study it was used as a positive control. Despite a comparatively low signal-to-background noise ratio of ∼5.6, Figure 4B demonstrates that ZO-1 proteins are appropriately located at the cell periphery in PNT2 cells.

When examining E-cadherin (Figure 4C), considerably enhanced signal-to-noise levels of over ∼33 are achieved. The superior quality of the images obtained is likely due to the brighter nature of the red quantum dots compared to green. Figure 4C reveals E-cadherin is primarily found along the PNT2 cell boundaries with particularly high levels of fluorescence concentrated in specific regions (circled in Figure 4C).

In order to examine regions with high fluorescent activity in closer detail, the boxed region in Figure 4A was subsequently scanned at higher-resolution. The resulting topography image (Figure 4E) reveals a network of filopodia from neighbouring cells that have established contact. The simultaneously acquired fluorescence image is shown in Figure 4D. A row of fluorescence corresponding to E-cadherin location can be seen running parallel to the edge of one cell (see arrow on Figure 4D). Additional rows of fluorescence are also present along the length of the large filopodium in the centre of the image (identified by arrowhead). These results confirm that the localisation of E-cadherin can be successfully examined in samples that have been dual stained.

### Structural Analysis of PC-3 cells

**Figure 5:**
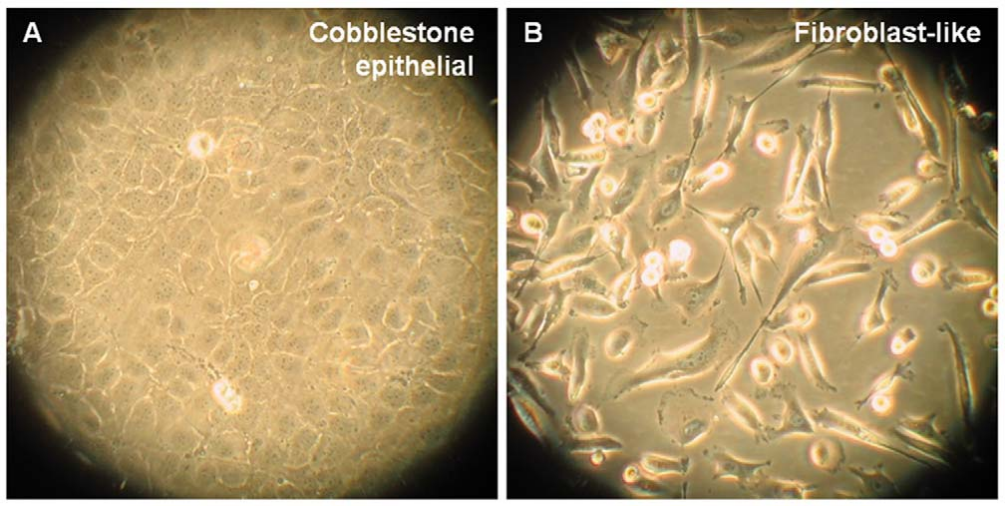
Morphology of (A) PNT2 cells and (B) PC-3 cells examined by bright field microscopy.

To compare the morphology of healthy and cancerous cells, bright field microscopy images were initially obtained. Confluent PNT2 cells are shown in Figure 5A, while PC-3 cells are shown in Figure 5B. The PC-3 cells display a markedly different appearance: some PC-3 cells display a spherical morphology, whereas others appear more elongated and fibroblast-like, often possessing elongated lamellipodia.

**Figure 6:**
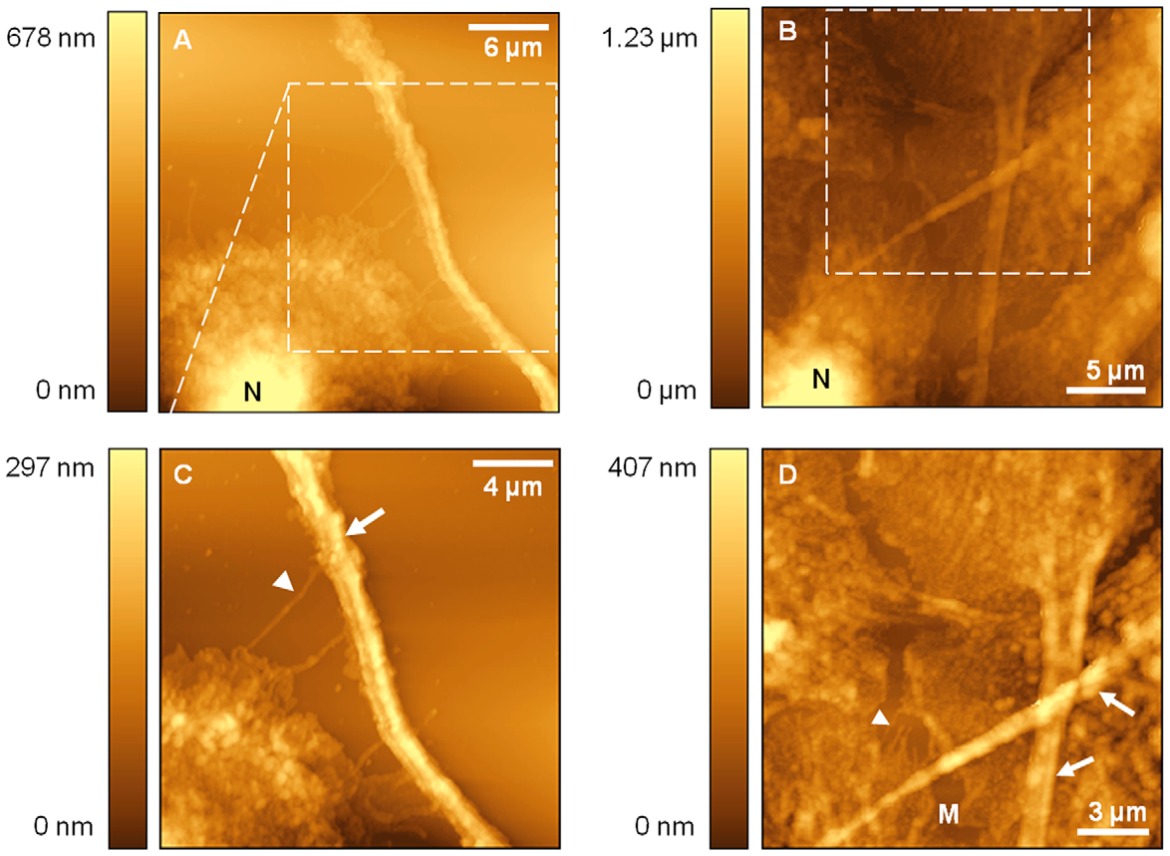
SNOM topography acquisitions of PC-3 cells. Image (A) is a typical example of a non-confluent PC-3 sample. The dotted square indicates a region that has been studied in further detail, which can be seen in image (C). In these images the PC-3 cells exhibit cytoplasmic protrusions, indicated by the arrow in image (C). Images (B) and (D) show a more confluent distribution of PC-3 cells. A zoomed region is outlined on image (B) and image (D) is the resultant zoom. The higher resolution clearly reveals loose cell-cell associations along the boundary between cells; this boundary is indicated by label M. In image (D) the cytoplasmic protrusions are again labelled with an arrow. Other labels used in the figures include arrowheads to indicate the filopodia locations and the label N is highlighting the cell nucleus.

Prior to immunolabelling of PC-3 cells, their morphology was further examined using high-resolution SNOM topography acquisitions. The images shown in Figure 6 directly confirm the observations of Figure 5. Some cells appear spherically shaped as demonstrated in Figure 6A while others exhibit a more fibroblast-like morphology with long, branching cytoplasmic protrusions (as indicated by arrows in Figures 6C and 6D). These protrusions are significantly longer than the filopodia typically observed on healthy cells and were often found to extend to lengths greater than 25–30 µm, i.e. beyond the size range of the topography scans. Additionally, measurements show their diameter to be much wider than the finer filopodia, some of which can be distinguished protruding from the lamellipodia as indicated by arrowheads in Figures 6C and 6D. These larger protrusions tend to taper as their length increases however, at their widest point they were found to measure up to 2.1 µm and to reach heights of up to approximately 300 nm. Measurements on the finer protrusions observed in these images revealed widths ranging between 120–340 nm. The cells' nuclei (indicated by N) can clearly be distinguished in Figures 6A and 6B. The boxed regions in Figures 6A and 6B represent areas that were subsequently zoomed in. This enabled superior contrast between features of similar height and exposed much more detail.

In contrast to confluent PNT2 cells, PC-3 cells do not appear to form very close associations with neighbouring cells. This is demonstrated by the images shown in Figure 6B and 6D. Gaps of up to 2.1 µm around the periphery of the cells are observed and no seal along the boundary between adjacent cells has been established (the cell-cell boundary is indicated by label M on the figure). Numerous fine filopodial extensions can be seen to protrude across these gaps; however few appear to interact with those from opposing cells. This is remarkably different to PNT2 cells where filopodia from adjacent cells were observed to interdigitate across the intercellular region. When PNT2 cells achieved high confluency, the cells were fully sealed together and no gaps could be distinguished.

### Dual labelled PC-3 cells

**Figure 7:**
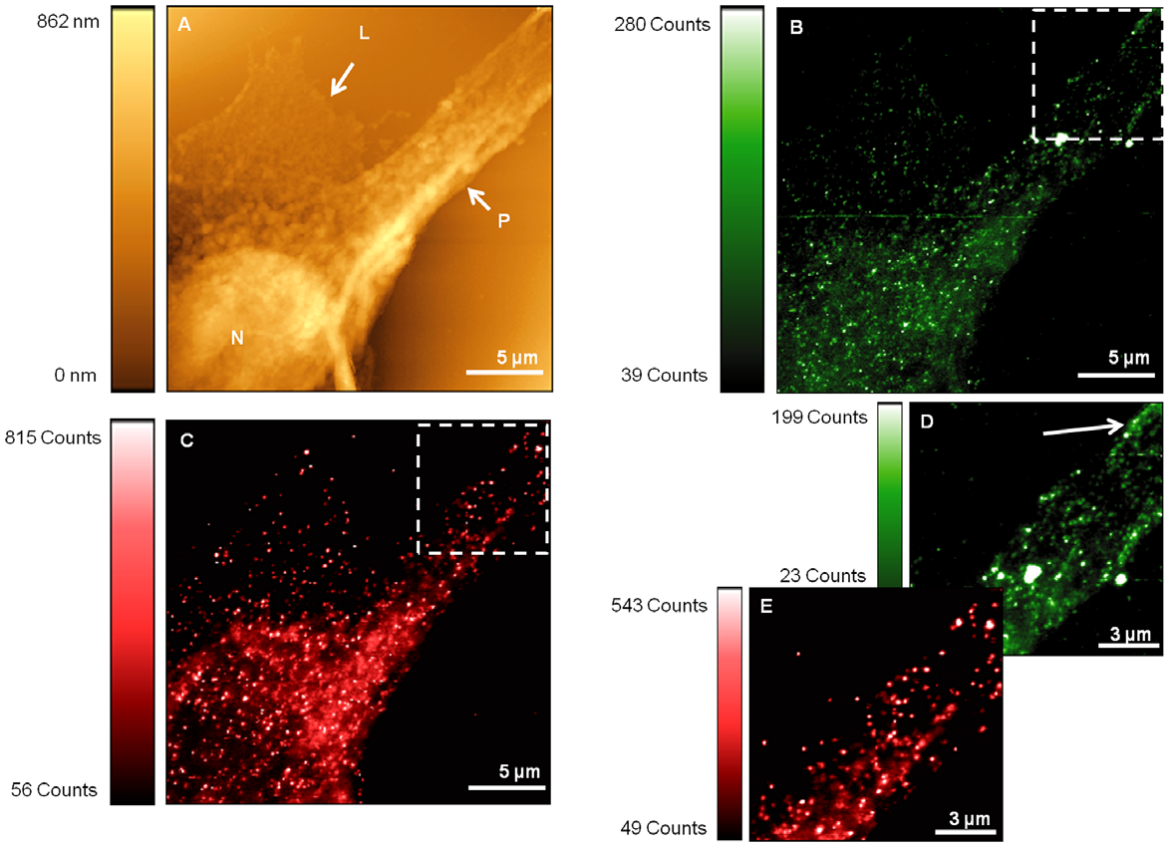
SNOM acquisitions of dual labelled PC-3 cells. Images reveal cell topography (A) and fluorescence (B, C, D and E). The nucleus is identified by N, lamellipodium by L and protrusion by P. ZO-1 localisation is shown in images (B and D) whilst E-cadherin labelling is shown in (C and E). The boxed regions in (B and C) correspond to the detailed zoom regions displayed in (D and E) respectively. The arrow in (D) highlights the superior periphery labelling of ZO-1.

In order to assess the sub-cellular localisation of E-cadherin protein in PC-3 cells, dual labelling was again undertaken. Typical SNOM acquisitions of dual labelled PC-3 cells are shown in Figure 7. The topographic image is shown in Figure 7A and reveals a single cell whose nucleus (labelled by N) is clearly distinguished. Measurements show that the maximum height of this cell is reached over the nuclear region which equates to approximately 890 nm. The lamellipodium (labelled by L) can be seen and was found to span an area of approximately 90 µm^2^. A large cellular protrusion is visible on the cell and is identified by the label P on the image. Measurements indicate a width of 6.2±0.1 µm at its point of origin.

Corresponding SNOM fluorescence acquisitions are shown in Figures 7B and 7C and were obtained using different optical band pass filters to distinguish between ZO-1 and E-cadherin respectively. A subsequent zoomed area (highlighted in Figure 7B and 7C) was generated by the software to enable the cell periphery to be resolved in more detail. Figures 7D and 7E reveal the relative labelling of ZO-1 and E-cadherin respectively.

As observed previously with the PNT2 cells, the signal-to-noise ratios achieved with the green quantum dots in Figure 7B are noticeably less compared to that achieved by red quantum dots in Figure 7C. The signal-to-noise ratio of Figure 7B is ∼7.2. Correlating the ZO-1 fluorescence image (Figure 7B) with the topography (Figure 7A) reveals fluorescence over the region of the cell that corresponds to the nucleus. However, fluorescence can also clearly be identified along the periphery of the cellular protrusion and is identified by arrows in the image shown in Figure 7D. Whilst the ZO-1 was primarily a tool for control, this re-distribution of fluorescence into the nuclear region (similar to the E-cadherin) has been seen in previous studies [20], [21].

Figure 7C shows the SNOM acquisition obtained to examine the localisation of E-cadherin proteins in PC-3 cells by recording the near-field fluorescence response of the red emitting quantum dot labelling. Although the image shows a strong fluorescence response from the sample, the signal-to-noise level (∼14.6) is notably reduced compared to that observed when examining E-cadherin in PNT2 cells. Moreover, fluorescence signals are evident predominantly around the perimeter of the nucleus and are also found to extend some way along the length of the protrusion. This can be seen more clearly in Figure 7E which represents a zoom of the boxed region in Figure 7C. Despite this response, there appears to be no significant E-cadherin labelling pattern around the periphery of the cell in the regions which confer cell-cell contact, as was seen with the PNT2 cell line. This gives a clear indication of a lack of appropriate protein localisation in the PC-3 cells.

## Discussion

Following previous investigations of adhesion mechanisms in the healthy epithelial cell line PNT2 [22], a dual labelling methodology has been developed to facilitate the study of adhesion molecules in other cell lines. Extensive optimisation of the protocols was necessary in order to generate high-quality fluorescent samples and to ensure that immunolabelling accurately reflects the distribution of proteins on the cell membrane. Many preparation aspects were taken into consideration during this procedure in order to achieve effective labelling with high signal response and low background labelling, as detailed in Materials and Methods.

Gene expression analysis revealed a significant down-regulation in E-cadherin expression in the PC-3 cell line compared to the control cell line, PNT2. This was validated at the protein level by Western blotting and is in agreement with other studies which show E-cadherin protein expression to be down regulated in PC-3 cells [23]. ZO-1 protein expression in both cell lines was found to be only slightly altered and thus could act as a suitable control signal. Hence a dual labelling approach was deemed necessary for investigations of E-cadherin localisation using SNOM as ZO-1 detection would ensure optical detection in our SNOM instrument was optimal, particularly in the event that E-cadherin signals could not be detected. To address the difficulties encountered during dual labelling of PC-3, the E-cadherin proteins were immunolabelled with brighter (owing to their higher quantum yield) red emitting quantum dots. Optimisation of the protocols was required to ensure that effective labelling was achieved with an accurate signal response. This was harder to achieve on the PC-3 cell line, as the E-cadherin expression is reduced and a poorly developed labelling protocol could simply be labelling the E-cadherin poorly. This in turn would present itself as reflecting a change of expression. From this extensive optimisation, dual labelling was successfully achieved and thus facilitated the study of the localisation of E-cadherin and ZO-1 proteins in two prostate epithelial cell lines using the high-resolution SNOM imaging.

Analysis of dual labelled healthy epithelial PNT2 cells revealed that E-cadherin is predominantly located along the cell periphery and appeared enhanced in regions where cell-cell contact was established. This was demonstrated by the punctate clusters of E-cadherin which were observed when filopodia established contact and initiated cell-cell adhesion as was shown in Figure 4C. PNT2 cells were found to form the typical cobblestone appearance that is characteristically seen in normal epithelial tissues. This was consistent with previous studies, where only the localisation of E-cadherin proteins was examined [22]. As expected, ZO-1 proteins were also found to be localised around the periphery of the PNT2 cells. This was clearly demonstrated in Figure 4B despite the quality of the ZO-1 fluorescence images being inferior to those obtained for E-cadherin, due to the quantum yield differences of the quantum dots used.

The study was extended to examine cancerous PC-3 cells. Initially the morphology of these metastatic cells was analysed through the acquisition of SNOM topography images. PC-3 cells were found to differ in structure to healthy epithelial cells in numerous ways. Firstly, their overall shape was found to be more irregular with some cells assuming a fibroblast-like morphology in comparison to the more cobblestone appearance of healthy cells. Secondly, PC-3 cells were often difficult to image due to their abrupt features. The PC-3 cells commonly possessed long cytoplasmic protrusions in addition to the filopodia routinely observed on healthy cells. Finally, examination of more confluent samples revealed that PC-3 cells form loose associations with neighbouring cells with few opposing filopodia interacting. Gaps were frequently observed around the periphery of the cells (as demonstrated by Figure 6D), in contrast to the tight seals that were observed during examination of PNT2 cells. The morphology of PC-3 cells observed in this study using SNOM confirms earlier observations made by Lang *et al.* [24] using phase contrast microscopy and scanning electron microscopy.

When imaging with SNOM, drawing quantitative comparisons of expression levels based on the number of counts recorded becomes difficult when different SNOM probes have been utilised. However, utilising the signal-to-noise ratio instead of the absolute counts helps to account for differences which may arise with a change of SNOM probe. Subsequent analysis of the sub-cellular localisation of E-cadherin and ZO-1 proteins in PC-3 cells was undertaken. Fluorescence SNOM acquisitions revealed the presence of ZO-1 on the periphery of cellular protrusions in addition to being observed in the nuclear region. Although not the primary focus of this study, this observation confirms the findings of other reports where delocalisation of ZO-1 is seen [21]. Indeed ZO-1 accumulation in the cell's nucleus in addition to cell-cell contact sites has been observed previously [20]. The signal-to-noise ratios measured when examining ZO-1 proteins is consistent across both cell lines studied and this supports the gene expression data and the Western blot analysis where little change in ZO-1 expression is seen.

Examination of SNOM fluorescence images revealed E-cadherin expression between the two cell lines to be distinctly different. In PNT2 the E-cadherin signals are dominant around the periphery of the cells, whereas in PC-3 cells E-cadherin is predominantly localised around the nuclear region of cells, with no obvious E-cadherin labelling pattern around the periphery. In addition, E-cadherin signal-to-noise ratios were analysed and the ratio was markedly reduced suggesting a deficiency of E-cadherin protein expression in addition to the lack of localisation at the periphery of PC-3 cells. This further supports the down regulation seen in gene expression data and Western blot analysis. However, neither of these two latter methods yield information about the location of E-cadherin, and consequently whether it is functional in its role as a cell-cell adhesion mediator. While fluorescence techniques have been employed in other studies in an effort to determine the localisation of E-cadherin in PC-3 cells [24], [25], the techniques utilised do not provide the high-resolution fluorescence and structural imaging afforded by SNOM. In these SNOM studies a clear difference has been observed in the morphology and phenotype between healthy PNT2 cells and cancerous PC-3 cells. The lack of appropriate E-cadherin expression in PC-3 cells can be directly linked to the lack of tight cell-cell associations with neighbouring cells in confluent samples.

Studies to consider the effects of a further protein, β-catenin are also of interest, particularly looking at its role for recruiting E-cadherin to the cell membrane and anchoring E-cadherin to the cytoskeleton. There is some evidence that β-catenin expression is also altered in both primary and metastatic prostate cancers [26], and further high resolution studies on β-catenin and additional adhesion proteins from various cell lines using these methods will give a fuller explanation of the mechanisms involved in metastatic cancer progression.
